# A case of sudden multiple hematomas during cesarean section due to amniotic fluid embolism

**DOI:** 10.1016/j.ijscr.2023.108342

**Published:** 2023-05-24

**Authors:** Shohei Tanabe, Akiko Yoshimoto, Sachiyo Sugino, Kotaro Ichida, Kiyoshi Niiya, Syuji Morishima

**Affiliations:** Kobe City Medical Center, West Hospital, Japan

**Keywords:** Amniotic fluid embolism, Cesarean section, Haematoma, Disseminated intravascular coagulation

## Abstract

**Introduction:**

We report a case of multiple hematomas, a rare manifestation of amniotic fluid embolism (AFE), during cesarean section.

**Presentation of case:**

The patient had a history of pregnancy and cesarean section birth due to placental abruption. At 38 weeks and 2 days, her membrane ruptured, and an emergency cesarean section was performed. During uterine suturing, hematomas suddenly formed in multiple locations, and bleeding began. Intraoperative blood tests revealed decreased hemoglobin and fibrinogen levels, leading to the administration of red blood cells and fresh frozen plasma. Despite initial transfusions, hemoglobin and fibrinogen levels did not increase, necessitating additional transfusions, which ultimately improved the hemoglobin and fibrinogen levels. A post-discharge blood draw showed decreased C3 levels, supporting a diagnosis of disseminated intravascular coagulation (DIC)-type AFE.

**Discussion:**

The sudden onset of hematomas in multiple locations other than the uterine incision wound was an atypical presentation of AFE in this case. The multiple hematomas were caused by DIC-induced hemostasis, and the decreased C3 level in the blood test supported the diagnosis of DIC-type AFE.

**Conclusion:**

Multiple hematomas may occur as a symptom of DIC-type AFE and require attention.

## Highlights

• The patient had multiple hematomas during cesarean section.
• The right ovary was removed during hematoma repair.
• Amniotic fluid embolism caused the bleeding.
• This case shows the risks of amniotic fluid embolism and the need for prompt diagnosis and management.

## 1 Introduction

Amniotic fluid embolism (AFE) is a rare but dangerous condition that can occur during or after delivery and is characterized by a sudden drop in blood pressure, acute hypoxia, and coagulopathy [1]. However, cases of AFE with atypical symptoms, such as chest pain and cold sensation, have also been reported [2]. In this report, we describe a patient with AFE who developed multiple hematomas and required right ovariectomy.

## 2 Case presentation

The patient was a 31-year-old G2P1 woman with a history of pregnancy and cesarean section of her first child due to early abruption of the placenta. The patient had no relevant medical history.

The pregnancy occurred spontaneously and progressed well. At 38 weeks and 2 days of pregnancy, an emergency cesarean section was performed because her membrane had ruptured. There were no problems until cesarean section was initiated and the baby was delivered. When uterine suturing was initiated, an intramuscular hematoma first formed on the anterior wall of the uterine body. A hematoma then developed next to the base of the uterus from the right uterine horn to the ovarian adnexa, and bleeding began. Repeated ligation failed to stop the bleeding, and the patient went into shock.

An intravenous route was added while blood was taken for testing, and fluid loading and continuous noradrenaline administration were initiated. Hematoma formation was evident from the right basilar ligament to the ovarian adnexa, and the adnexa had nearly ruptured. Blood tests submitted intraoperatively revealed decreased levels of hemoglobin (Hb), 5.3 g/dl; platelets, 113,000/μl; and fibrinogen, 184 mg/dl. Emergency transfusion of 6 units of Type O red blood cells (RBC) and 4 units of AB fresh frozen plasma (FFP) was initiated. The hematoma was incised, and the right adnexa was removed. Marked bleeding stopped, but complete hemostasis was not achieved. Complete hemostasis could not be achieved without improving coagulation function through blood transfusion, and the operation was terminated.

The total blood loss was 1869 ml, including the amniotic fluid. Blood tests after the transfusion showed Hb, 5.5 g/dl; platelets, 85,000/μl; and fibrinogen, 92.8 mg/dl, which did not improve, and an additional 8 units of RBC and 10 units of FFP were administered. After the additional administration, blood tests showed improvement in the levels of Hb, 9.0 g/dl; platelets, 67,000/μl; and fibrinogen, 232 mg/dl. Subsequently, the patient's condition showed good improvement; she was discharged on the 8th postoperative day.

Immediately after delivery, the patient experienced a massive hemorrhage of unknown cause; as no other explanation for the causative disease was available, a diagnosis of clinical AFE was made. Laboratory tests submitted to the Amniotic Fluid Embolism Group of Hamamatsu Medical School, which conducts clinical research on AFE, revealed the following: zinc coproporphyrin-1 (Zn-CP1), < 1/6 p mol/l; sialyl Tn antigen (STN), 23.0 U/ml < 45.0 U/ml; decreased C3 levels, 76.0 mg/dl < 80 mg/dl; C4, 14.0 mg/dl > 11.0 mg/dl; and C1 esterase inhibitors, 61.0 % > 42.0 %. Thus, uterine-type AFE is considered to be the cause of massive hemorrhage and disseminated intravascular coagulation (DIC). The resected right adnexa showed no abnormal pathological findings. She was discharged and sent home eight days after surgery.

## 3 Discussion

Amniotic fluid embolism is a dangerous disease that can lead to maternal death. Previous reports have indicated that the incidence of fatal AFE is 0.99 per 100,000, making early diagnosis and treatment important [3]. In the present case, fortunately for the patient, blood tests were performed intraoperatively to anticipate the possibility of AFE; this enabled the early initiation of blood transfusion.

The sudden onset of hematomas in multiple locations other than the uterine incision wound was an atypical presentation of AFE in this case. The case of AFE with DIC and a subcapsular hematoma [4] and the number of reports of hematomas in soft tissue due to coagulopathy [5] suggest that the multiple hematomas in the uterus in this case were caused by DIC-induced hemostasis. The cause of the multiple uterine hematomas in the present case may have been hemostatic failure due to DIC.

Compared to patients with postpartum hemorrhage, patients with AFE have been reported to have severe coagulopathy and significantly lower fibrinogen levels, despite significantly less blood loss at onset [6]. In this report, the correlation with AFE was strongest when the cutoff value for the fibrinogen level was 132 mg/dl, and the fact that the fibrinogen level also decreased to 92.8 mg/dl in this case may reflect the decreased fibrinogen level with the onset of AFE. Previous reports have characterized low fibrinogen levels as a cause of significant postpartum hemorrhage [7]. Moreover, fibrinogen concentrate has been reported to be more useful than FFP when fibrinogen levels are deficient [8]. Ideally, although we should have used fibrinogen concentrate in this case as well, we could not because it was not available at our hospital.

A definitive diagnosis of AFE is made based on autopsy findings of fetal squamous cells in the maternal pulmonary arterial blood [9]. However, an autopsy was obviously not performed in this case. Other serum markers for AFE, such as Zn-CP1, STN, and complement C3, C4, and C1 esterase inhibitors have been reported as serum markers for AFE by a group at the Hamamatsu University School of Medicine in Japan. As serum markers, the levels of Zn-CP1 and STN are increased in cardiopulmonary collapse-type AFE, while the levels of C3 and C4 are decreased in DIC-type AFE [10]. As the current patient did not experience cardiac arrest or severe hypoxia and because symptoms were consistent with DIC, the decreased C3 level in the blood test supported DIC-type AFE.

## 4 Conclusion

Some cases of AFE develop suddenly during cesarean section, with multiple hematomas as the initial symptom. In such cases, AFE treatment should be initiated promptly.

## Declaration of competing interest

The authors state no conflict of interest.

## Data Availability

Additional data is available via reasonable request made to the corresponding author.
